# Uveitis: Molecular Pathogenesis and Emerging Therapies

**DOI:** 10.3389/fimmu.2021.623725

**Published:** 2021-04-30

**Authors:** Charles E. Egwuagu, Sahar A. Alhakeem, Evaristus C. Mbanefo

**Affiliations:** ^1^ Molecular Immunology Section, Laboratory of Immunology, National Eye Institute (NEI), National Institutes of Health, Bethesda, MD, United States; ^2^ Department of Biomedical Sciences, College of Health Sciences, University of Wisconsin-Milwaukee, Milwaukee, WI, United States

**Keywords:** molecular therapies, cellular therapies, autoimmunity, immunobiology, EAU, uveitis

## Abstract

The profound impact that vision loss has on human activities and quality of life necessitates understanding the etiology of potentially blinding diseases and their clinical management. The unique anatomic features of the eye and its sequestration from peripheral immune system also provides a framework for studying other diseases in immune privileged sites and validating basic immunological principles. Thus, early studies of intraocular inflammatory diseases (uveitis) were at the forefront of research on organ transplantation. These studies laid the groundwork for foundational discoveries on how immune system distinguishes self from non-self and established current concepts of acquired immune tolerance and autoimmunity. Our charge in this review is to examine how advances in molecular cell biology and immunology over the past 3 decades have contributed to the understanding of mechanisms that underlie immunopathogenesis of uveitis. Particular emphasis is on how advances in biotechnology have been leveraged in developing biologics and cell-based immunotherapies for uveitis and other neuroinflammatory diseases.

## Introduction

The vertebrate immune system is comprised of the adaptive and innate immune systems and the two limbs have unique as well as overlapping functions that are seamlessly integrated (1). During antigen priming in secondary lymphoid or peripheral tissues, innate immune cells such as dendritic cells (DC) secrete cytokines that instruct the naïve lymphocyte to differentiate and generate effector lymphocyte subsets that orchestrate humoral or cell-mediated immunity to pathogens (2). The immense capacity of lymphocyte response to diverse antigens derives from diversity of their cell surface antigen receptors and a critical function of the immune system is to establish tolerance against self-antigens while allowing selective immunity to pathogens (3, 4). Thus, exuberant effector immune responses that cause pathogenic autoimmunity are restrained or prevented by specialized regulatory cells that secrete immune-suppressive cytokines (5). Defects in central and/or peripheral tolerance mechanisms can result in autoimmune diseases such as uveitis, the focus of this review. Uveitis is a group of potentially blinding intraocular inflammatory diseases of infectious or autoimmune etiology and accounts for more than 10% of severe visual handicaps in the United States (6, 7). Although steroids and other anti-inflammatory drugs are effective therapies, renal toxicity and other adverse effects preclude their prolonged usage. Development of effective and safe therapies thus requires a better understanding of molecular and cellular mechanisms that maintain ocular immunity and how dysregulation of these pathways contribute to pathogenesis of uveitis. In this review, we highlight recent advances in understanding of mechanisms that underlie susceptibility to uveitis and summarize emerging therapeutic approaches that show promise in suppressing ocular inflammation and uveitis.

## The Vertebrate Eye

**Figure 1:**
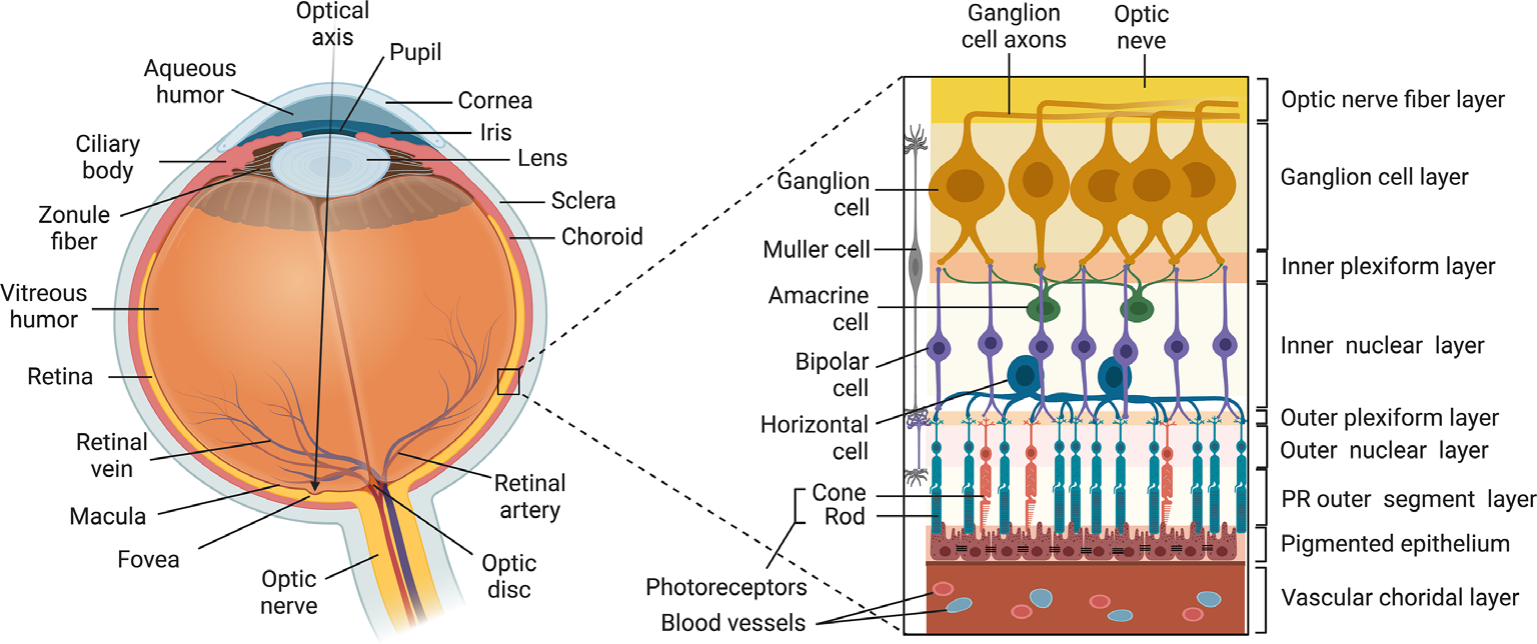
Structure of the Vertebrate Eye. The eye is composed of: (i) the outer tunic of the eye comprised of the sclera and cornea (ii) the uvea comprised of the choroid, the iris and the ciliary body; (iii) the neural retina comprised of five types of neurons (ganglion cell, amacrine cells, bipolar cells, horizontal cells and photoreceptors (rods and cones); (iv) Muller cell; (v) RPE.

The eye is segregated from the peripheral immune system and is arguably one of the most anatomically complex organs in mammals (**Figure 1**). It is composed of: (i) the outer coat of the eye comprised of the opaque sclera (outer white layer of the eyeball) and the transparent cornea which is a continuation of the sclera; (ii) The retinal pigment epithelium (RPE) is a single layer of hexagonal pigmented cells overlying the retina and attached to the underlying choroid. It functions in phagocytosis of photoreceptor outer segment membranes, light absorption, epithelial transport and nourishment of visual cells of the neuroretina. (iii) The Mueller/Glia cell is a specialized glial cell type that supports and supplies nutrients and oxygen to retinal neurons and serves in maintaining the functional stability of retinal cells. The Mueller cell also insulates neurons from each other, uptakes neurotransmitters and regulates the extracellular environment. (iv) The uvea is the pigmented middle layer of the eye beneath the cornea and sclera and is comprised of the vascularized choroid, iris and ciliary body. It performs most visual functions of the eye including focusing on objects at various distances to the retina and changing the pupil size in response to light intensity. (v) The neural retina is the innermost, light-sensitive layer consisting of five types of neurons that receive photons transmitted through the cornea and lens. It consists of the photosensitive ganglion cells, amacrine cells, bipolar cells, horizontal cells and photoreceptors (rods and cones). They generate two-dimensional image of an object and converts it to electrical signals that are then transduced to the brain to create visual perception. The embryonic vertebrate retina and optic nerve derive from the diencephalon of the developing brain and are considered part of the CNS. Similar to brain, the retina is an immune privileged tissue.

## The Eye as an Immune Privileged Site

Developments in ocular immunology led to appreciation of the special relationship between the eye and the immune system and this foundational research led to our current concept of immune privilege. Sir Peter Medawar who shared the 1960 Nobel Prize in physiology and medicine with Sir Frank Macfarlane Burnet coined the term “Immune Privilege” based on studies showing that tumor or skin allograft when placed in the anterior chamber of the eye is not rejected (8). Immune privilege was then viewed as a unique feature of the eye and failure to reject an allograft or alloantigen was attributed to the lack of blood vessels in retina (9). A modern explanation of immune privilege is that proteins in immune privileged tissues of the CNS are sequestrated from the peripheral immune system by the blood-retina barrier (BRB), blood-brain-barrier (BBB) and the neurovascular unit (NVU) comprised of pericytes, perivascular macrophages, neuronal dendrites, glia limitans of the Müler/microglia and tightly bound endothelial cells that surround the NVU (10, 11). The avascular immunosuppressive environment of the retina lacks lymphatic drainage, contains resident regulatory cells that secrete neuropeptides and anti-inflammatory cytokines and also contributes to immunological sequestration of the retina (10). In addition, the RPE and resident retinal cells that express inhibitory cell surface associated proteins (TGF-β, FAS/FAS ligand, CD46 and CD59) limit inflammation in the retina by inactivating lymphocytes (12–14). Normal development of the gut microbiome during early infancy is now recognized to play important roles in establishing the BBB or BRB (15) and studies in mice with defective gut microbiome have established a link between dysbiosis and susceptibility to inflammatory diseases including, acute anterior uveitis (AAU) (16, 17). Despite these safeguards, intense and persistent inflammation can still overwhelm the multilayered barriers and peripheral tolerance mechanisms that maintain immune privilege. Th17 cells play important roles of in early events that initiate pathogenesis of inflammatory diseases (18–20). They produce granzyme B that promotes disruption of the BBB and initiate CNS autoimmune diseases including multiple sclerosis and experimental autoimmune encephalomyelitis (EAE) (21). Similar to other CNS autoimmune diseases, adoptive transfer studies have shown that as few as 1x10^7^ uveitogenic Th1 and Th17 cells also promote breakdown of the BRB and the retinal NVU, resulting in the recruitment of bystander lymphocytes and myeloid cells that exacerbate uveitis (20). However, individuals with intact immune system eventually re-establish immune privilege *via* induction of regulatory T cells (Tregs, Tr1, Tr3) and B cells (Bregs) that secrete immune-suppressive cytokines in the retina and lymphoid tissues.

## Human Uveitis

CNS inflammatory diseases present unique challenges because the need to eliminate a pathogen is as important as avoiding exuberant inflammatory response associated with photoreceptor cell deficit and development of severe uveitis. A cross-sectional study in California documented an incidence rate of 25.6 /100,000 person-years and prevalence rate of 69 cases/100,000 persons (22). However, infectious uveitis accounts for less than 20% of uveitis/scleritis, with incidence rate of 18.9/100,000 and prevalence of 60.6/100,000 persons (23). Uveitis is classified as anterior, intermediate, posterior or pan uveitis depending on the anatomic location (24). Anterior segment uveitis is the most common form and manifests as iritis or iridocyclitis while Intermediate uveitis is characterized by vitritis and peripheral retinal vasculitis. Posterior uveitis is a disease of the posterior segment (retina, choroid and vitreous) and symptoms include blurred vision, photophobia, retinal neovascularization, retinal detachment and macular edema. Although the precise etiology of most uveitis is difficult to ascertain, Fuchs’ heterochromic iridocyclitis, birdshot retinochoroidopathy, multifocal choroiditis, pars planitis and sympathetic ophthalmia are thought to be of autoimmune etiology (24). Uveitis can also be associated with systemic diseases such as sarcoidosis, psoriatic arthritis, ankylosing spondylitis, juvenile rheumatoid arthritis (JRA), MS, Vogt-Koyanagi-Harada’s disease, Behçet’s disease, systemic lupus erythematosus (SLE) and collagen vascular diseases (24).

## Animal Models of Uveitis

Animal models of human uveitis have contributed to understanding of immunopathogenic mechanisms of uveitis. Two of the best characterized models of anterior uveitis are endotoxin-induced uveitis (EIU) (25) and experimental autoimmune anterior uveitis (EAAU), also called experimental melanin-induced uveitis (EMIU) (26, 27). Posterior uveitis poses the greatest risk of blindness and the best characterized model of posterior uveitis is experimental autoimmune uveitis (EAU) (28–30). Study of EAU using a variety of genetically modified mouse strains have identified critical pathways that mediate posterior uveitis. These studies have identified transcription factors (STAT3, IRF4, IRF8), regulatory proteins (SOCS1, SOCS3) or cytokine signaling pathways that regulate EAU and can serve as potential therapeutic targets for ameliorating uveitis (20, 31–35). Of particular interest is a transgenic mouse strain expressing a TCR specific for IRBP_161−180_ that develops spontaneous ocular autoimmunity. Analysis of EAU in these mice has revealed that gut commensals might be a source of signals that prime autoreactive retina-specific T cells to trigger uveitis (36).

### Experimental Models of Anterior Uveitis

Endotoxin-induced uveitis (EIU) is a rodent model of acute anterior segment inflammation induced by subcutaneous or intraperitoneal injection of lipopolysaccharide (LPS) and is characterized by infiltration of inflammatory cells into the anterior segment (37). Although EIU exhibits immunohistopathologic features of human anterior uveitis, duration of the inflammatory response is relatively short (< 72h) and does not cause lasting tissue damage (37). On the other hand, EAAU is induced by peripheral administration of proteins bound to melanin granules and the inflammatory response is more representative of human anterior uveitis (26, 27, 38). EAAU is characterized by massive infiltration of mononuclear and polymorphonuclear cells into the anterior chamber, iris and ciliary body vessels, with limited posterior segment involvement. Although none of these models manifest full spectrum of clinical and histopathological features of human uveitis, each contributes to understanding of particular aspects of the disease process.

### Experimental Autoimmune Uveitis: Model of Posterior Uveitis

Experimental autoimmune uveitis (EAU) is a T-cell-mediated intraocular inflammatory disease induced in susceptible species by active immunization with ocular-specific proteins or peptides in an emulsion containing Complete Freund’s Adjuvant (CFA). Full-blown disease requires coadministration of pertussis toxin and heat-killed tuberculosis bacteria, which activate bacterial pattern recognition receptors on innate immune cells (28). Commonly used retinal protein for inducing EAU in mice and rats are S-Antigen (S-Ag or arrestin) and interphotoreceptor retinoid-binding protein (IRBP) (39, 40). EAU is considered a useful model of posterior uveitis because of its immunopathologic features that include iritis, choroiditis, vitritis, retinal vasculitis, destruction of photoreceptor cells and retinal edema (20). The disease is transferable to naive syngeneic animals by injection of *in vitro* activated CD4^+^ T cells specific to retinal antigens (28–30).

## Cellular and Molecular Mechanisms That Determine Susceptibility or Resistance to Uveitis

Although lymphocytes from uveitis patients respond to retinal S-Ag *in vitro* and a number of retinal antigens induce disease in rodents and non-human primates, the putative retinal antigens involved in human uveitis have not been defined. On the other hand, it has been suggested that the response to S-Ag might be secondary to retinal tissue damage induced by inflammation. Nevertheless, animal models of uveitis exhibit essential immunopathogenic features of human uveitis. In this section of the review, we summarize what we know of the molecular pathogenesis of uveitis with particular focus on: molecular basis of susceptibility to autoimmune uveitis and source of the autoreactive memory T cells that mediate relapsing-remitting inflammation that characterize potentially blinding chronic uveitis.

### Defects in Central Tolerance Mechanism and Susceptibility to Uveitis

**Figure 2:**
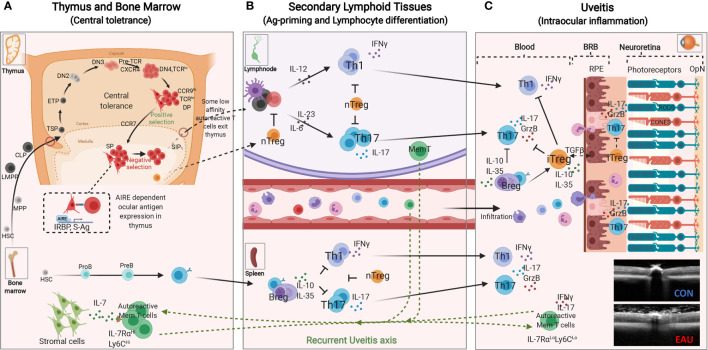
Immunopathogenic Mechanisms of experimental autoimmune uveitis (EAU). **(A)** Central tolerance mechanism: Bone marrow derived lymphoid-primed multipotent progenitors (LMPPs) and common lymphoid progenitors (CLPs) enter the thymus near the cortico-medullary junction. Thymus-settling progenitors cells give rise to early T cell progenitors (ETPs), double negative 1 (DN1), DN2 and DN3 thymocytes that then migrate to the subcapsular zone for further development (42). DN3 thymocytes that express functional pre-T cell receptor and CXCR4 receive survival signals that promote proliferation and eventual differentiate to DN4 and then double positive (DP) thymocytes. The DP thymocytes (CCR9^Hi^) undergoing positive selection interact with self-peptide/MHC complexes on cortical thymic epithelial cells, upregulate CCR7 and mature into single positive (SP) mature T cells that migrate to the thymic medulla (42). The medullary thymic epithelial cells (mTEC) in collaboration with the AIRE transcription factor (autoimmune regulator) in the medulla, promiscuously express tissue-restrictive antigens of major proteins in peripheral tissues. AIRE also contributes to mechanism of negative selection, which eliminates self-reactive T cells that would cause autoimmune diseases. T cells with normal low affinity/avidity recognition of self-antigens are induced to upregulate sphingosine-1-phosphate receptor 1 (S1P1), exit the thymus and enter the blood and peripheral lymphoid tissues. **(B)** Peripheral tolerance mechanisms mediated by nTregs render potentially autoreactive T cells anergic or “ignorant”. Naïve T cells that enter the circular or peripheral tissues differentiate to various T-helper subsets in response to PAMPs (pathogen associated molecular patterns) or molecular mimicry mechanism. During EAU, active immunization with ocular antigens (e.g. IRBP or S-Ag) in CFA emulsion induces clonal expansion of Th1 and Th17 resulting in disease by day 14-20 followed by Treg and Breg-mediated resolution of the acute inflammatory response between days 25-32 after disease induction. However, few autoreactive memory T cells expressing IL-7Rα persist and they eventually migrate to the bone marrow (BM) where they reside and can be reactivated to mediate recurrent uveitis. **(C)** Schematic representation of early events associated with loss of immune privilege of the eye and induction of retinal protective mechanism in rodent model of uveitis. Effector molecules such as Granzyme B and proinflammatory cytokines secreted by Th17 cells facilitate breakdown of blood retina barrier (BRB), resulting in the influx of other inflammatory cells such as Th1, Th2, and monocytes. The inflammatory cells entering the eye encounter hostile environment of the neuroretina consisting of anti-inflammatory molecules as well as regulatory T and B cells secreting IL-10 and/or IL-35. RPE, retinal pigment epithelium; OPN, optic nerve; CON, control retina; EAU, OCT image of mouse with uveitis.

The mature T lymphocyte derives from bone marrow hematopoietic stem cells (HSC) and acquires capacity to mediate immunological responses following maturation in the thymus. At very early stages of its development, bone marrow-derived lymphoid-primed multipotent progenitor (LMPP) and common lymphoid progenitor (CLP) enter the thymic cortex and undergo positive and negative selection processes (central tolerance) that endows the developing T cell with the capacity to discriminate between self and non-self-antigens (41, 42). T cells that express a functional TCR and develop tolerance for self-antigens encountered in the thymus exit the thymus to enter secondary lymphoid organs and the peripheral circulation (**Figure 2**). The AIRE transcription factor (autoimmune regulator) plays a critical role in central tolerance mechanism by promoting the expression of self-proteins on medullary thymic epithelial cells (mTEC) at levels detectable by the maturing T cells. T cells with very high affinity for cognate self-protein are eliminated because they pose danger of pathogenic autoimmunity (1, 43). However, some T cells that detect cognate antigens just below the threshold required for tolerance induction are not eliminated and these renegade autoreactive T cells are thought to be the cause of organ-specific autoimmune diseases (44). In immune-competent hosts, these potentially autoreactive T cells are maintained at low levels by an immune-suppressive T cell subset that also develops in the thymus (45). These specialized Foxp3^+^ T cells are called natural regulatory T cells (nTregs) and they constitutively secrete IL-10, an immune-suppressive cytokine that suppresses autoreactive T cells and inflammation (5). These nTregs also migrate from the thymus into peripheral tissues and play critical roles in peripheral tolerance.

A major contribution of ocular immunology and uveitis to our current understanding of the molecular basis of resistance or susceptibility to organ-specific autoimmune diseases derived from a series of EAU studies performed in the late nineties. Genetic and molecular studies of the thymus of mouse strains (B10RIII, B10A, C57BL/6 and BALB/c) and rat strains (Lewis and Brown-Norway) that vary in their relative susceptibility to EAU provided direct evidence that ocular proteins are not sequestered from the peripheral immune system and are accessible for tolerance induction in the thymus (43). These studies showed that ocular proteins are expressed in the thymus, but the level of expression varied even among animals of the same species including mice, rats and non-human primates (43, 46, 47). High level expression of ocular proteins (IRBP or S-Antigen) in the thymus correlated with resistance to EAU development while low levels correlated with susceptibility to uveitis. This observation was subsequently validated in a mouse model of PLP-induced EAE (48) and in human studies showing that transcription levels of the insulin gene in the thymus correlated with susceptibility to type 1 diabetes (49). This seminal discovery from EAU studies thus provided mechanistic explanation for differential susceptibility to autoimmune uveitis and suggested that resistance to uveitis is regulated at least in part by capacity to establish central tolerance to retinal autoantigens (43).

### Pro-inflammatory T-Helper Cells Mediate Acute and Chronic Relapsing-Remitting Uveitis

Uveitis correlates with HLA class I or class II genes, with HLA-DR4 strongly associated with sympathetic ophthalmia or VKH while HLA-A29 is strongly associated with birdshot retinochoroidopathy (50, 51). On the other hand, HLA-B27 is strongly associated with anterior uveitis and patients with acute anterior uveitis and ankylosing spondylitis (52). Similar to humans, susceptibility of mice to uveitis is linked to certain genetic loci, with strong correlations to certain MHC class II haplotypes (53). Among mouse strains the most susceptible is B10.RIII with decreasing susceptibility: B10.RIII (H-2r)> B10.BR (H-2k) >C57BL/6 (H-2b) (53). However, the genetic influences of the MHC molecules are not the only predisposing factors.

The heterodimeric IL-12 family of cytokines play critical roles during Ag-presentation in developmental decisions of differentiating naïve lymphocytes and in determining the intensity, duration and nature of the inflammatory response (54). The family is comprised of IL-12 (IL-12p35/IL-12p40), IL-23 (IL-23p19/IL-12p40), IL-27 (IL-27p28/Ebi3), IL-35 (IL-12p35/Ebi3), and IL-39 (IL-23p19/Ebi3) (54, 55). Studies in the late nineties demonstrated that IL-12 skewed the uveitogenic T cell response towards the Th1 developmental pathway while IL-12p40-deficient mice were found to be resistant to EAU, leading to the suggestion that Th1 cells mediated EAU (56). Subsequent discovery of IL-23 that shares the IL-12p40 subunit with IL-12 led to appreciation of the role of IL-23 in several autoimmune diseases and eventual identification of the Th17 as the lymphocyte subset that mediated these diseases (19). IL-12 was subsequently shown to confer protection against EAU (57), leading to the revised conclusion that IL-23 rather than IL-12 is critical for the development of EAU induced by immunization with IRBP in CFA (58). Soon after the discovery of Th17, its role in EAU was established (59). This was confirmed by studies showing that mutant mice with targeted deletion of STAT3 in the CD4 T cell compartment do not develop EAU because their T cells cannot differentiate into Th17 cells (31). The EAU-resistant CD4-STAT3KO mice exhibited exaggerated increase of Th1 cells and elevated levels of IFN-γ, providing suggestive evidence that increase in Th1 cells does not cause EAU (31). Subsequent studies revealed that Th1 expansion during EAU antagonized Th17 responses through IFN-γ/STAT1-dependent expression of IL-27 cytokine, an immune-suppressive member of the IL-12 family (59). It is notable that involvement of Th17 cells in a human autoimmune disease was first demonstrated in study of uveitis and scleritis patients. Blood of patients with active uveitis or scleritis contained elevated levels of Th17 which correlated with increase in IL-2 (59).

Organ-specific CNS autoimmune diseases such as uveitis and multiple sclerosis are characterized by repeated cycles of remission and recurrent inflammation and it is not known where the auto-reactive memory T cells that initiate recurrent uveitis or MS reside during periods of disease remission. This age-old question was addressed in a chronic uveitis model by monitoring IRBP-specific autoreactive memory T cells (IL-7Rα^High^Ly6C^High^CD4^+^) for 6 months and showing that the uveitogenic T cells relocated from the retina and peripheral lymphoid tissues into the bone marrow (BM) (20) (**Figure 2**). The autoreactive memory T cells resided in a resting state in the BM and upon restimulation converted to Th17 effector cells (IL-7Rα^Low^Ly6C^Low^CD4^+^). It is notable that while BM cells from WT EAU mice transferred uveitis to naive mice, IRBP-specific autoreactive memory T cells of CD4-STAT3KO mice could not traffic to the BM and BM cells from the IRBP-immunized CD4-STAT3KO mice could not transfer EAU upon reactivation (20). These studies identified BM as a niche for IRBP-specific memory T cells that caused recurrent uveitis and suggested that BM stromal cells provide survival signals to autoreactive memory T cells through STAT3-dependent mechanisms (20). Taken together, analysis of mouse uveitis model and the blood of human uveitis patients identified roles of Th17 cells and STAT3 pathway in uveitis and that STAT3 pathway is a potential target for treatment of uveitis.

## Treatment of Uveitis

Topical or systemic steroids are effective therapies for uveitis. While topical corticosteroid is effective for anterior uveitis, severe intermediate or posterior uveitis might require periocular corticosteroid injections. On the other hand, standard of care for vision-threatening uveitis, especially if accompanied by cystoid macular edema is systemic immunosuppression with oral corticosteroid (prednisone). If ineffective, low dose prednisone in combination with cyclosporin A, antimetabolites (methotrexate, azathioprine) or anti-inflammatory (colchicine) is indicated. When combination of corticosteroid with these second line drugs are ineffective, alkylating agents such as cyclophosphamide or chlorambucil which are associated with significant adverse effects are employed as a last resort. Nonetheless, prolonged use of systemic corticosteroids increases risk of infections, malignancy and decreased lifespan. Thus, the adverse effects of prolonged use of corticosteroids, alkylating agents and immunosuppressive drugs such as cyclosporin A, FK-506 or rapamycin are impetus to develop less toxic and more specific therapies for uveitis.

## Posterior Uveitis therapy

### Targeting Cytokines and Cytokine Receptors

Commonly used therapies for posterior uveitis are steroids and although they are effective in suppressing ocular inflammation, they do not directly target memory autoreactive T cells that perpetuate cycles of recurrence and remission that characterize blinding posterior uveitis.

Besides steroids, biologics that target T cell receptors or effector functions are gaining acceptance although Adalimumab (Humira) is the only FDA approved biologic for uveitis. Other biologics include: (i) Blockage of T cell signaling pathways (cyclosporine, FK-506 and rapamycin); (ii) Anti-CD4 T cell function (anti-IL-2R, anti-IFN-γ Abs; (iii) Targeting TNF-α (Etanercept (Enbrel^®^), Infliximab (Remicade^®^), Thalidomide); (iv) Biologics that target immune modulatory molecules (adhesion, co-stimulatory molecules) (60–63). This section of the review will focus on new therapeutic strategies that target signal transduction pathways utilized by pathogenic lymphocytes.

### Targeting Janus Kinases

The JAK/STAT pathway regulates growth and survival of mammalian cells and signal transduction through this evolutionarily conserved pathway is mediated by Janus kinases (Jak1, Jak2, Jak3 and Tyk2) and Signal Transducers and Activators of Transcription (STAT1, STAT2, STAT3, STAT4, STAT5a, STAT5b, and STAT6), a 7-member family of latent cytoplasmic transcription factors (64). Immunoregulatory cytokines secreted by innate cells (IL-12, IL-23, IL-27) or lymphocytes (IFN-γ, IL-2, IL-4, IL-6, IL-10, IL-21) mediate their biological activities through receptor-associated Janus kinases (Jaks) and Stat proteins (65). Cytokine-binding to its cognate receptor selectively activates Jaks by transphosphorylation of specific tyrosine residues, followed by recruitment of cytokine-specific Stats to the receptor complex (66). Tyrosine-phosphorylated Stats form homo- or hetero-dimers, translocate into the nucleus and mediate transcription of cognate genes. Thus, cytokine-mediated activation of Jak/Stat pathway provides a rapid membrane to nucleus mechanism for regulating gene expression and altering behavior of the cell (66). Because persistent activation of Jak/Stat signals dysregulate the immune system and cause many autoimmune diseases and cancer, cytokine responses are under stringent regulation. Thus, pharmacological regulation of Jak kinase activities has been exploited for treatment of autoimmune and neoplastic diseases (67–71). Of note, Jak inhibitors have been found to be effective in treating anterior uveitis and associated macular edema (72).

#### Targeting Stat3 Pathway Utilized by Th17 Cells

Most cytokines that regulate lymphocyte growth, development, survival or effector functions activate STAT3 pathway (73, 74). CD4-STAT3KO mice do not develop EAU because of defect in generating Th17 cells (31), suggesting that STAT3 is a potential therapeutic target for modulating uveitis. Surprisingly, mice with loss of STAT3 in the CD19 B cell compartment (CD19-STAT3KO) have exacerbate EAU (32), suggesting that intrinsic functions of STAT3 are diametrically opposite in T and B cells. Another target of Stat3 that promotes ocular inflammation is miR-155-5p (miR-155). Stat3 activates miR-155 and the Stat3/miR-155 axis mediate severe uveitis by promoting the expansion of pathogenic Th17 cells (75). Inflammatory cytokines have also been shown to induce expression of miR-155 in human RPE cells (76), further suggesting that Stat3 is a potential therapeutic target for treating uveitis. We describe here strategies that have been successful in targeting Stat pathway and suppressing uveitis in mice.

#### Synthetic STAT3 Inhibitors

**Figure 3:**
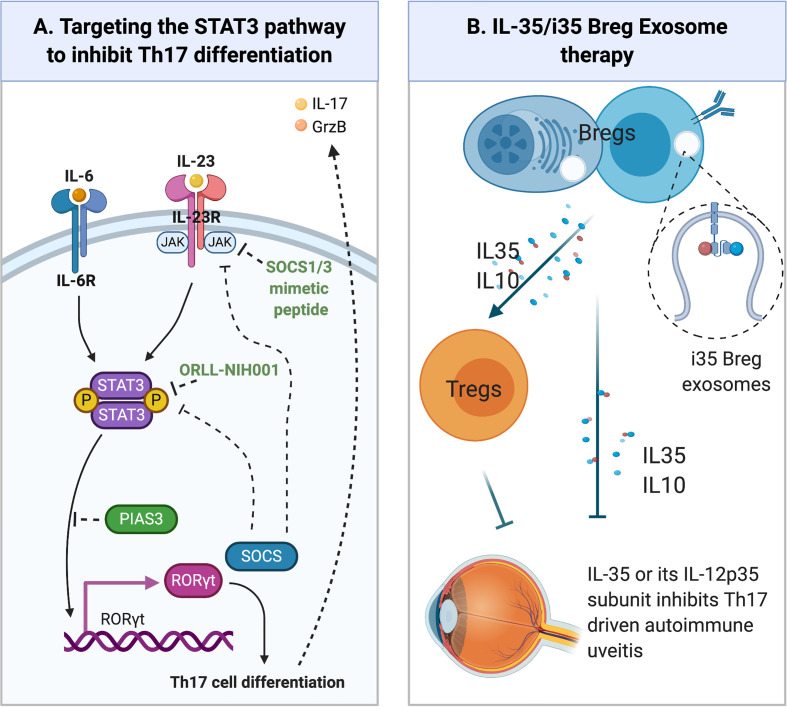
Emerging Therapies for Uveitis. **(A)** Regulation of STAT3 pathway. Cytokines such as IL-6 or IL-23 bind their cognate receptor on lymphocytes and activate receptor-associated JAK kinases. Following the recruitment of latent STAT3 proteins to the activated receptor complex, the STAT3 protein is Tyrosine-phosphorylated, forms pSTAT3:pSTAT3 homodimers that translocate to the nucleus and activate STAT3-responsive genes. SOCS (SOCS1, SOCS3) proteins are immediate early genes activated by pSTAT3 and they are negative feedback regulators of JAK/STAT pathway. They inhibit or terminate JAK/STAT signals by binding to Tyrosine-phosphorylated JAKs or cytokine receptors, targeting them for degradation in the proteosome. PIAS3 protein also inhibits STAT3 transcriptional activities by binding STAT3 DNA binding domain and physically preventing STAT3 binding to target genes. **(B)** Other emerging therapies for the treatment of uveitis include: (i) Immunotherapy with IL-35-producing Breg cells (i35-Breg) (ii) Administration of biologics (IL-35, IL12p35); (iii) Exosome treatment with IL-35-containing exosomes (i35-Exosomes).

ORLL-NIH001 is a synthetic 406-kDa small chemical compound that targets STAT3 (**Figure 3**) by antagonizing Th17 cells, down-regulating α4;β1, α4β7, CCR6 and CXCR3 and inhibiting trafficking of lymphocytes into the retina (77). However, a drawback to therapeutic use of ORLL-NIH001 is its relatively poor bioavailability and frequent administration of the drug is required. Although delivery of the drug by intravenous injection is effective, oral administration or subcutaneous injection may be attractive alternatives.

### Suppressor of Cytokine Signaling and SOCS Mimetic Peptide Therapy

Suppressor of cytokine signaling (SOCS) are an 8-member family of intracellular proteins that are rapidly induced in many cell types in response to cytokines (IFN-γ, IL-2, IL-4, IL-6, IL-10, IL-12, IL-21, IL-23, IL-27) or growth factors (CNTF, LIF, FGF, IGF-1, insulin). However, unabated stimulation of Stat signaling pathway during chronic inflammation or cellular stress induces SOCS proteins, particularly SOCS1 and SOCS3 that function as components of a negative feedback loop that regulate the initiation, intensity and duration of inflammatory cytokine responses (78). Their inhibitory effects derive from direct interaction with cytokine/growth-factor receptors, STAT proteins and Jak kinases, leading to proteasomal degradation of target proteins or receptor complex and termination of the signal (79, 80). SOCS1 and SOCS3 are well established as important regulators of inflammatory diseases including allergy, autoimmune diseases, diabetes, metabolic syndrome and cancer (79, 80). In context of potential involvement of SOCS proteins in uveitis, transgenic rats and mice with targeted overexpression of SOCS1 in the retina are protected from severe uveitis and scleritis patients exhibit a defect in SOCS1 expression as their lymphocytes could not induce SOCS1 in response to IL-2 (81). These observations suggest that enhancing SOC1 levels in the retina can be used to suppress uveitis. However, a major impediment to use of SOCS1 or SOCS3 for therapy is that they are intracellular proteins and the lack of efficient means to deliver these proteins into cells. A promising new approach is the development of SOCS1 and SOCS3 mimetic peptides that incorporate an N-terminal or carboxy-terminal lipid moiety, which then allows SOCS mimetic peptides to be delivered directly into cells.

#### Topical SOCS1 Mimetic Peptide Therapy

The SOCS1 protein possesses a kinase inhibitory region (KIR) and binding of the KIR to activated Jak1 or Jak2 suppresses their kinase activities (82). SOCS1 mimetic peptide of varying length have been shown to be effective in inhibiting Stat1/Stat3 signaling (83, 84). Topical administration of a formulation consisting of membrane-penetrating 16 amino acid SOCS1 peptide, with a lipophilic palmitoyl-lysine group attached to its NH2-terminus (SOCS1-KIR), was effective in suppressing uveitis in mice EAU (**Figure 3**) (85). The SOCS1-Mimetic ameliorated uveitis by suppressing the expansion of pathogenic Th17 cells and trafficking of inflammatory cells into the neuroretina during EAU. Photopic and scotopic electroretinograms confirmed the neuroprotective effects of the SOCS1-KIR in uveitis (85). Importantly, the SOCS1-Mimetic is non-toxic, indicating that topical administration of SOCS1-Mimetics can be exploited as a non-invasive treatment for uveitis.

## Emerging Therapies for Uveitis

### Therapeutic Cytokines

The IL-12 family of cytokines is comprised of proinflammatory (IL-12, IL-23, IL-39) and immunosuppressive members (IL-27 and IL-35) (54). In mouse models of uveitis, the increase of IL-12 or IL-23 during antigen priming in LN or the eye promotes uveitis (86) while increased secretion of IL-27 by microglial cells or lymphocytes during EAU suppressed EAU (59, 87, 88). These reports suggest that identifying factors that induce the immune-suppressive IL-27 and IL-35 cytokines or enhance their biological activities *in vivo* might be potential therapeutic targets that can be exploited to suppress human ocular inflammatory diseases.

### Interleukin 35 Therapy

Following discovery that IL-35-producing Treg cells (iT_R_35) suppressed colitis in the mouse (89), Wang et al. showed that treatment of mice with recombinant IL-35 suppressed EAU by inhibiting Th-17 responses and inducing expansion of regulatory B cells (Breg) that produce IL-10 (B10) or IL-35 (i35-Bregs) (90). Adoptive transfer of *ex-vivo* generated i35-Bregs cells was also shown to suppress EAU, providing evidence that i35-Breg immunotherapy may also be efficacious in uveitis (90) (**Figure 3**). Mechanistically, the i35-Bregs also suppressed EAU by inhibiting expansion of pathogenic Th17 cells and converting conventional lymphocytes to B10, iTR35 or i35-Breg. These observations have led to significant interest in using IL-35 or i35-Bregs as treatment for uveitis. However, before i35-Breg immunotherapy can be brought to the clinic, additional preclinical investigations are needed to establish IL-35 pharmacokinetics and detailed characterization of human i35-Breg to demonstrate that it is a stable Breg phenotype with immune-suppressive activity and functions.

### IL-35 Subunit Therapy

Despite significant interest in IL-35 as a potential biologic or use of i35-Breg immunotherapy for autoimmune diseases, less is known about mechanisms by which IL-35 mediates its immune suppressive functions. It is still not clear whether immune-suppressive activities of IL-35 derive exclusively by the pairing of IL-12p35 and Ebi3 subunits to form the heterodimeric IL-35 or if IL-12p35 or Ebi3 also possesses intrinsic functions independent of IL-35. Recent reports revealed that recombinant IL-12p35 (rIL-12p35) has immunoregulatory activities that regulated immunity and suppressed neuroinflammation in the EAE mouse model (91). Similar to IL-35, the rIL-12p35 subunit suppressed EAU and EAE by inducing expansion of B10 and i35-Bregs while inhibiting Th17 responses (91, 92). However, a major difference is that while IL-35 upregulates inhibitory receptor proteins (Lag3, PD-1) that induce T cell exhaustion, rIL-12p35 did not induce these inhibitory receptors but suppressed inflammation by inhibiting signals downstream of IL-6 receptor and cell-cycle proteins that inhibit T cell proliferation (91). Recapitulation of essential immunosuppressive activities of IL-35 suggests that IL-12p35 may also be used as immunotherapy for uveitis.

### i35-Breg-Exosome Therapy

Although IL-35 shows promise as biologics for the treatment of autoimmune diseases, a major disadvantage of using the heterodimeric IL-35 cytokine for therapy is its instability and relatively short-half-life. Besides the technical challenges associated with *ex-vivo* generation of large amounts of functional i35-Breg, there is the difficulty of reproducibly administering the same dose of IL-35 each time. It is a very unstable heterodimeric cytokine because association of its IL-12p35 and Ebi3 subunits is not strong (non-covalent) and readily dissociate. This makes it difficult to ascertain the dose of the bioactive p35:Ebi3 heterodimer administered or required to ameliorate disease. An important development relating to the therapeutic use of IL-35 or i35-Breg is the recent discovery that i35-Bregs secrete copious amounts of exosomes that contain IL-35 (93). An impediment to i35-Breg immunotherapy for neuroinflammatory diseases are the BBB, BRB and NVU that limit entry of cells into the CNS. Exosomes are 30-150 nm nanosized extracellular vesicles and their relatively small size, making them suitable for delivering immunoregulatory molecules to the CNS. Mice treated with IL-35 containing exosomes (i35-Exosomes) were protected from developing severe uveitis and disease protection correlated with expansion of IL-10 and IL-35 secreting Treg cells with concomitant suppression of Th17 responses (93) (**Figure 3**). i35-Exosome therapy thus overcomes the technically difficult and labor-intensive effort required to produce sufficient amounts of IL-35-producing i35-Bregs for immunotherapy: as much as 32x10^10^ exosomes can be isolated from a mouse and 2x10^10^ i35-exosomes contain ~15ng IL-35. Another advantage of i35-Exosomes therapy is that IL-12p35/Ebi3 heterodimers are confined in the same vesicle which obviates the dosing issue of determining the precise amount of bioactive IL-35 administered to the subject (93). Taken together, i35-Exosomes are an attractive therapeutic option for delivering IL-35 to the retina.

## Perspectives

The remarkable advances in ocular immunology over the past 3 decades have led to a better understanding of the molecular mechanisms that underlie etiology and susceptibility to uveitis. Genetically modified mouse strains with targeted deletion or overexpression of transcription factors, regulatory proteins or cytokines that control lymphocyte development or functions have led to identification of critical pathways that regulate uveitis. Results from these studies have ushered in a new era of targeted therapies for these family of potentially blinding diseases that are a major cause of morbidity. In conclusion, we highlight three promising and effective treatment modalities without the adverse effects associated with steroids, which are the commonly used drugs for uveitis. (i) Regulatory B cell (Breg) therapy: Breg immunotherapy with as few as 5x10^6^ cells is sufficient to suppress uveitis by inhibiting Th17/Th1 lymphocytes and converting conventional lymphocytes into IL-10 and/or IL-35-producing regulatory cells. Because the Bregs proliferate *in vivo* they are able to sustain secretion of anti-inflammatory cytokines in target tissues. (ii) i35-Exosome therapy: i35-Exosomes suppress uveitis by upregulating inhibitory receptors (PD1, LAG3), propagating infectious-tolerance signals which induced conventional lymphocytes to acquire capacity for producing immunosuppressive cytokines. (iii) Topical SOCS1-Mimetic therapy: The SOCS1-Mimetic inhibits Jak kinases and is an effective non-invasive treatment for uveitis in mice. Although therapy for uveitis will continue to depend in part upon the location, underlying cause and any associated complications, availability of alternative therapeutic options for patients is clearly a major dividend.

## Author Contributions

CE, SA, and EM wrote and reviewed manuscript. All authors contributed to the article and approved the submitted version.

## Conflict of Interest

The authors declare that the research was conducted in the absence of any commercial or financial relationships that could be construed as a potential conflict of interest.

## References

[1] Gery I Egwuagu CE. Central Tolerance Mechanisms in Control of Susceptibility to Autoimmune Uveitic Disease. Int Rev Immunol (2002) 21(2-3):89–100. 10.1080/08830180212061 12424838

[2] Takenaka MC Quintana FJ. Tolerogenic Dendritic Cells. Semin Immunopathol (2017) 39(2):113–20. 10.1007/s00281-016-0587-8 PMC5296314 27646959

[3] Theofilopoulos AN Kono DH Baccala R. The Multiple Pathways to Autoimmunity. Nat Immunol (2017) 18(7):716–24. 10.1038/ni.3731 PMC5791156 28632714

[4] Amaya-Uribe L Rojas M Azizi G Anaya JM Gershwin ME. Primary Immunodeficiency and Autoimmunity: A Comprehensive Review. J Autoimmun (2019) 99:52–72. 10.1016/j.jaut.2019.01.011 30795880

[5] Sakaguchi S Mikami N Wing JB Tanaka A Ichiyama K Ohkura N. Regulatory T Cells and Human Disease. Annu Rev Immunol (2020) 38:541–66. 10.1146/annurev-immunol-042718-041717 32017635

[6] Gritz DC Wong IG. Incidence and Prevalence of Uveitis in Northern California; the Northern California Epidemiology of Uveitis Study. Ophthalmology (2004) 111(3):491–500; discussion 500. 10.1016/j.ophtha.2003.06.014 15019324

[7] Caspi RR. A Look At Autoimmunity and Inflammation in the Eye. J Clin Invest (2010) 120(9):3073–83. 10.1172/JCI42440 PMC2929721 20811163

[8] Medawar PB. Immunity to Homologous Grafted Skin; the Fate of Skin Homografts Transplanted to the Brain, to Subcutaneous Tissue, and to the Anterior Chamber of the Eye. Br J Exp Pathol (1948) 29(1):58–69. 18865105 PMC2073079

[9] Medawar PB. Immunological Tolerance. Science (1961) 133(3449):303–6. 10.1126/science.133.3449.303 13768822

[10] Molzer C Heissigerova J Wilson HM Kuffova L Forrester JV. Immune Privilege: The Microbiome and Uveitis. Front Immunol (2020) 11:608377. 10.3389/fimmu.2020.608377 33569055 PMC7868421

[11] Forrester JV McMenamin PG Dando SJ. CNS Infection and Immune Privilege. Nat Rev Neurosci (2018) 19(11):655–71. 10.1038/s41583-018-0070-8 30310148

[12] Kawazoe Y Sugita S Keino H Yamada Y Imai A Horie S. Retinoic Acid From Retinal Pigment Epithelium Induces T Regulatory Cells. Exp Eye Res (2012) 94(1):32–40. 10.1016/j.exer.2011.11.002 22116001

[13] Zamiri P Masli S Streilein JW Taylor AW. Pigment Epithelial Growth Factor Suppresses Inflammation by Modulating Macrophage Activation. Invest Ophthalmol Vis Sci (2006) 47(9):3912–8. 10.1167/iovs.05-1267 16936104

[14] Zamiri P Masli S Kitaichi N Taylor AW Streilein JW. Thrombospondin Plays a Vital Role in the Immune Privilege of the Eye. Invest Ophthalmol Vis Sci (2005) 46(3):908–19. 10.1167/iovs.04-0362 15728547

[15] Braniste V Al-Asmakh M Kowal C Anuar F Abbaspour A Toth M. The Gut Microbiota Influences Blood-Brain Barrier Permeability in Mice. Sci Transl Med (2014) 6(263):263ra158. 10.1126/scitranslmed.3009759 PMC4396848 25411471

[16] Rosenbaum JT. New Developments in Uveitis Associated With HLA B27. Curr Opin Rheumatol (2017) 29(4):298–303. 10.1097/BOR.0000000000000403 28376061

[17] Rosenbaum JT Asquith M. The Microbiome and HLA-B27-associated Acute Anterior Uveitis. Nat Rev Rheumatol (2018) 14(12):704–13. 10.1038/s41584-018-0097-2 PMC6597169 30301938

[18] Boivin WA Cooper DM Hiebert PR Granville DJ. Intracellular Versus Extracellular Granzyme B in Immunity and Disease: Challenging the Dogma. Lab Invest (2009) 89(11):1195–220. 10.1038/labinvest.2009.91 PMC7102238 19770840

[19] Cua DJ Sherlock J Chen Y Murphy CA Joyce B Seymour B. Interleukin-23 Rather Than interleukin-12 is the Critical Cytokine for Autoimmune Inflammation of the Brain. Nature (2003) 421(6924):744–8. 10.1038/nature01355 12610626

[20] Oh HM Yu CR Lee Y Chan CC Maminishkis A Egwuagu CE. Autoreactive Memory CD4+ T Lymphocytes That Mediate Chronic Uveitis Reside in the Bone Marrow Through STAT3-dependent Mechanisms. J Immunol (2011) 187(6):3338–46. 10.4049/jimmunol.1004019 PMC3304102 21832158

[21] Kebir H Kreymborg K Ifergan I Dodelet-Devillers A Cayrol R Bernard M. Human TH17 Lymphocytes Promote Blood-Brain Barrier Disruption and Central Nervous System Inflammation. Nat Med (2007) 13(10):1173–5. 10.1038/nm1651 PMC5114125 17828272

[22] Suhler EB Lloyd MJ Choi D Rosenbaum JT Austin DF. Incidence and Prevalence of Uveitis in Veterans Affairs Medical Centers of the Pacific Northwest. Am J Ophthalmol (2008) 146(6):890–896 e898. 10.1016/j.ajo.2008.09.014 19027424

[23] Zhang Y Amin S Lung KI Seabury S Rao N Toy BC. Incidence, Prevalence, and Risk Factors of Infectious Uveitis and Scleritis in the United States: A Claims-Based Analysis. PloS One (2020) 15(8):e0237995. 10.1371/journal.pone.0237995 32841267 PMC7447056

[24] Nussenblatt RB. The Natural History of Uveitis. Int Ophthalmol (1990) 14(5-6):303–8. 10.1007/BF00163549 2249907

[25] Rosenbaum JT McDevitt HO Guss RB Egbert PR. Endotoxin-Induced Uveitis in Rats as a Model for Human Disease. Nature (1980) 286(5773):611–3. 10.1038/286611a0 7402339

[26] Broekhuyse RM Kuhlmann ED Winkens HJ Van Vugt AH. Experimental Autoimmune Anterior Uveitis (EAAU), a New Form of Experimental Uveitis. I. Induction by a Detergent-Insoluble, Intrinsic Protein Fraction of the Retinal Pigment Epithelium. Exp Eye Res (1991) 52(4):465–74. 10.1016/0014-4835(91)90044-f 2037026

[27] Broekhuyse RM Kuhlmann ED Winkens HJ. Experimental Autoimmune Anterior Uveitis (EAAU). III. Induction by Immunization With Purified Uveal and Skin Melanins. Exp Eye Res (1993) 56(5):575–83. 10.1006/exer.1993.1071 8500566

[28] Wacker WB Donoso LA Kalsow CM Yankeelov JA Jr. Organisciak DT. Experimental Allergic Uveitis. Isolation, Characterization, and Localization of a Soluble Uveitopathogenic Antigen From Bovine Retina. J Immunol (Baltimore Md. (1977) 119(6):1949–58. 334977

[29] Caspi RR Roberge FG Chan CC Wiggert B Chader GJ Rozenszajn LA. A New Model of Autoimmune Disease. Experimental Autoimmune Uveoretinitis Induced in Mice With Two Different Retinal Antigens. J Immunol (Baltimore Md. (1988) 140(5):1490–5. 3346541

[30] McAllister CG Wiggert B Chader GJ Kuwabara T Gery I. Uveitogenic Potential of Lymphocytes Sensitized to Interphotoreceptor Retinoid-Binding Protein. J Immunol (1987) 138(5):1416–20. 3805723

[31] Liu X Lee YS Yu CR Egwuagu CE. Loss of STAT3 in CD4+ T Cells Prevents Development of Experimental Autoimmune Diseases. J Immunol (2008) 180(9):6070–6. 10.4049/jimmunol.180.9.6070 PMC2435016 18424728

[32] Oladipupo FO Yu CR Olumuyide E Jittaysothorn Y Choi JK Egwuagu CE. STAT3 Deficiency in B Cells Exacerbates Uveitis by Promoting Expansion of Pathogenic Lymphocytes and Suppressing Regulatory B Cells (Bregs) and Tregs. Sci Rep (2020) 10(1):16188. 10.1038/s41598-020-73093-1 33004854 PMC7529787

[33] Kim SH Burton J Yu CR Sun L He C Wang H. Dual Function of the IRF8 Transcription Factor in Autoimmune Uveitis: Loss of IRF8 in T Cells Exacerbates Uveitis, Whereas Irf8 Deletion in the Retina Confers Protection. J Immunol (2015) 195(4):1480–8. 10.4049/jimmunol.1500653 PMC4530071 26163590

[34] Yu CR Hayashi K Lee YS Mahdi RM Shen de F Chan CC. Suppressor of Cytokine Signaling 1 (SOCS1) Mitigates Anterior Uveitis and Confers Protection Against Ocular HSV-1 Infection. Inflammation (2015) 38(2):555–65. 10.1007/s10753-014-9962-6 PMC4282977 24993154

[35] Egwuagu CE Sztein J Mahdi RM Li W Chao-Chan C Smith JA. IFN-Gamma Increases the Severity and Accelerates the Onset of Experimental Autoimmune Uveitis in Transgenic Rats. J Immunol (1999) 162(1):510–7. 9886427

[36] Horai R Caspi RR. Microbiome and Autoimmune Uveitis. Front Immunol (2019) 10:232. 10.3389/fimmu.2019.00232 30837991 PMC6389708

[37] McMenamin PG Crewe J. Endotoxin-Induced Uveitis. Kinetics and Phenotype of the Inflammatory Cell Infiltrate and the Response of the Resident Tissue Macrophages and Dendritic Cells in the Iris and Ciliary Body. Invest Ophthalmol Vis Sci (1995) 36(10):1949–59. 7657537

[38] Jha P Sohn JH Xu Q Nishihori H Wang Y Nishihori S. The Complement System Plays a Critical Role in the Development of Experimental Autoimmune Anterior Uveitis. Invest Ophthalmol Vis Sci (2006) 47(3):1030–8. 10.1167/iovs.05-1062 PMC1975680 16505038

[39] Zigler JS Jr. Mochizuki M Kuwabara T Gery I. Purification of Retinal S-antigen to Homogeneity by the Criterion of Gel Electrophoresis Silver Staining. Invest Ophthalmol Vis Sci (1984) 25(8):977–80. 6204954

[40] Gery I Wiggert B Redmond TM Kuwabara T Crawford MA Vistica BP. Uveoretinitis and Pinealitis Induced by Immunization With Interphotoreceptor Retinoid-Binding Protein. Invest Ophthalmol Vis Sci (1986) 27(8):1296–300. 3488297

[41] Hogquist KA Baldwin TA Jameson SC. Central Tolerance: Learning Self-Control in the Thymus. Nat Rev Immunol (2005) 5(10):772–82. 10.1038/nri1707 16200080

[42] Love PE Bhandoola A. Signal Integration and Crosstalk During Thymocyte Migration and Emigration. Nat Rev Immunol (2011) 11(7):469–77. 10.1038/nri2989 PMC3710714 21701522

[43] Egwuagu CE Charukamnoetkanok P Gery I. Thymic Expression of Autoantigens Correlates With Resistance to Autoimmune Disease. J Immunol (1997) 159(7):3109–12. 9317106

[44] Roep BO. Autoreactive T Cells in Endocrine/Organ-Specific Autoimmunity: Why has Progress Been So Slow? Springer Semin Immunopathol (2002) 24(3):261–71. 10.1007/s00281-002-0109-8 12503054

[45] Kronenberg M Rudensky A. Regulation of Immunity by Self-Reactive T Cells. Nature (2005) 435(7042):598–604. 10.1038/nature03725 15931212

[46] Ham DI Fujimoto C Gentleman S Chan CC Yu CR Yu S. The Level of Thymic Expression of RPE65 Inversely Correlates With its Capacity to Induce Experimental Autoimmune Uveitis (EAU) in Different Rodent Strains. Exp Eye Res (2006) 83:897–902. 10.1016/j.exer.2006.04.013 16777093

[47] Takase H Yu CR Mahdi RM Douek DC Dirusso GB Midgley FM. Thymic Expression of Peripheral Tissue Antigens in Humans: A Remarkable Variability Among Individuals. Int Immunol (2005) 17(8):1131–40. 10.1093/intimm/dxh275 PMC2366090 16030131

[48] Klein L Klugmann M Nave KA Tuohy VK Kyewski B. Shaping of the Autoreactive T-cell Repertoire by a Splice Variant of Self Protein Expressed in Thymic Epithelial Cells. Nat Med (2000) 6(1):56–61. 10.1038/71540 10613824

[49] Pugliese A Zeller M Fernandez A Jr. Zalcberg LJ Bartlett RJ Ricordi C. The Insulin Gene is Transcribed in the Human Thymus and Transcription Levels Correlated With Allelic Variation At the INS Vntr-IDDM2 Susceptibility Locus for Type 1 Diabetes. Nat Genet (1997) 15(3):293–7. 10.1038/ng0397-293 9054945

[50] Nussenblatt RB Mittal KK Ryan S Green WR Maumenee AE. Birdshot Retinochoroidopathy Associated With HLA-A29 Antigen and Immune Responsiveness to Retinal s-Antigen. Am J Ophthalmol (1982) 94(2):147–58. 10.1016/0002-9394(82)90069-1 6956239

[51] Gocho K Kondo I Yamaki K. Identification of Autoreactive T Cells in Vogt-Koyanagi-Harada Disease. Invest Ophthalmol Vis Sci (2001) 42(9):2004–9. 11481264

[52] Yang P Wan W Du L Zhou Q Qi J Liang L. Clinical Features of HLA-B27-positive Acute Anterior Uveitis With or Without Ankylosing Spondylitis in a Chinese Cohort. Br J Ophthalmol (2018) 102(2):215–9. 10.1136/bjophthalmol-2016-309499 28607176

[53] Caspi RR Grubbs BG Chan CC Chader GJ Wiggert B. Genetic Control of Susceptibility to Experimental Autoimmune Uveoretinitis in the Mouse Model. Concomitant Regulation by MHC and non-MHC Genes. J Immunol (1992) 148(8):2384–9. 1560198

[54] Vignali DA Kuchroo VK. Il-12 Family Cytokines: Immunological Playmakers. Nat Immunol (2012) 13(8):722–8. 10.1038/ni.2366 PMC4158817 22814351

[55] Wang X Wei Y Xiao H Liu X Zhang Y Han G. A Novel IL-23p19/Ebi3 (Il-39) Cytokine Mediates Inflammation in Lupus-like Mice. Eur J Immunol (2016) 46(6):1343–50. 10.1002/eji.201546095 PMC11334612 27019190

[56] Tarrant TK Silver PB Chan CC Wiggert B Caspi RR. Endogenous IL-12 is Required for Induction and Expression of Experimental Autoimmune Uveitis. J Immunol (Baltimore Md. (1998) 161(1):122–7. 9647215

[57] Tarrant TK Silver PB Wahlsten JL Rizzo LV Chan CC Wiggert B. Interleukin 12 Protects From a T Helper Type 1-Mediated Autoimmune Disease, Experimental Autoimmune Uveitis, Through a Mechanism Involving Interferon Gamma, Nitric Oxide, and Apoptosis. J Exp Med (1999) 189(2):219–30. 10.1084/jem.189.2.219 PMC2192986 9892605

[58] Luger D Caspi RR. New Perspectives on Effector Mechanisms in Uveitis. Semin Immunopathol (2008) 30:135–43. 10.1007/s00281-008-0108-5 PMC2756230 18317764

[59] Amadi-Obi A Yu CR Liu X Mahdi RM Clarke GL Nussenblatt RB. T(H)17 Cells Contribute to Uveitis and Scleritis and are Expanded by IL-2 and Inhibited by IL-27/STAT1. Nat Med (2007) 13(6):711–8. 10.1038/nm1585 17496900

[60] Takeuchi M. A Systematic Review of Biologics for the Treatment of Noninfectious Uveitis. Immunotherapy (2013) 5(1):91–102. 10.2217/imt.12.134 23256801

[61] Podojil JR Miller SD. Targeting the B7 Family of Co-Stimulatory Molecules: Successes and Challenges. BioDrugs (2013) 27(1):1–13. 10.1007/s40259-012-0001-6 PMC3653133 23329394

[62] Lechner MG Russell SM Bass RS Epstein AL. Chemokines, Costimulatory Molecules and Fusion Proteins for the Immunotherapy of Solid Tumors. Immunotherapy (2011) 3(11):1317–40. 10.2217/imt.11.115 PMC3226699 22053884

[63] Ford ML Adams AB Pearson TC. Targeting Co-Stimulatory Pathways: Transplantation and Autoimmunity. Nat Rev Nephrol (2014) 10(1):14–24. 10.1038/nrneph.2013.183 24100403 PMC4365450

[64] Darnell JE Jr. Stats and Gene Regulation. Science (1997) 277(5332):1630–5. 10.1126/science.277.5332.1630 9287210

[65] Egwuagu CE Larkin Iii J. Therapeutic Targeting of STAT Pathways in CNS Autoimmune Diseases. Jakstat (2013) 2(1):e24134. 10.4161/jkst.24134 24058800 PMC3670276

[66] Levy DE Darnell JE Jr. Stats: Transcriptional Control and Biological Impact. Nat Rev Mol Cell Biol (2002) 3(9):651–62. 10.1038/nrm909 12209125

[67] Leonard WJ Lin JX O’Shea JJ. The Gammac Family of Cytokines: Basic Biology to Therapeutic Ramifications. Immunity (2019) 50(4):832–50. 10.1016/j.immuni.2019.03.028 30995502

[68] Kontzias A Kotlyar A Laurence A Changelian P O’Shea JJ. Jakinibs: A New Class of Kinase Inhibitors in Cancer and Autoimmune Disease. Curr Opin Pharmacol (2012) 12(4):464–70. 10.1016/j.coph.2012.06.008 PMC3419278 22819198

[69] Laurence A Pesu M Silvennoinen O O’Shea J. Jak Kinases in Health and Disease: An Update. Open Rheumatol J (2012) 6:232–44. 10.2174/1874312901206010232 PMC3460320 23028408

[70] O’Shea JJ Kontzias A Yamaoka K Tanaka Y Laurence A. Janus Kinase Inhibitors in Autoimmune Diseases. Ann Rheum Dis (2013) 72 Suppl 2:ii111–115. 10.1136/annrheumdis-2012-202576 23532440 PMC3616338

[71] O’Shea JJ Schwartz DM Villarino AV Gadina M McInnes IB Laurence A. The JAK-STAT Pathway: Impact on Human Disease and Therapeutic Intervention. Annu Rev Med (2015) 66:311–28. 10.1146/annurev-med-051113-024537 PMC5634336 25587654

[72] Bauermann P Heiligenhaus A Heinz C. Effect of Janus Kinase Inhibitor Treatment on Anterior Uveitis and Associated Macular Edema in an Adult Patient With Juvenile Idiopathic Arthritis. Ocul Immunol Inflammation (2019) 27(8):1232–4. 10.1080/09273948.2019.1605453 31268748

[73] Egwuagu CE. STAT3 in CD4+ T Helper Cell Differentiation and Inflammatory Diseases. Cytokine (2009) 47(3):149–56. 10.1016/j.cyto.2009.07.003 PMC2733795 19648026

[74] Liu X Nurieva RI Dong C. Transcriptional Regulation of Follicular T-helper (Tfh) Cells. Immunol Rev (2013) 252(1):139–45. 10.1111/imr.12040 PMC3579502 23405901

[75] Escobar T Yu CR Muljo SA Egwuagu CE. STAT3 Activates miR-155 in Th17 Cells and Acts in Concert to Promote Experimental Autoimmune Uveitis. Invest Ophthalmol Vis Sci (2013) 54(6):4017–25. 10.1167/iovs.13-11937 PMC3680004 23674757

[76] Kutty RK Nagineni CN Samuel W Vijayasarathy C Hooks JJ Redmond TM. Inflammatory Cytokines Regulate microRNA-155 Expression in Human Retinal Pigment Epithelial Cells by Activating JAK/STAT Pathway. Biochem Biophys Res Commun (2010) 402(2):390–5. 10.1016/j.bbrc.2010.10.042 PMC2992362 20950585

[77] Yu CR Lee YS Mahdi RM Surendran N Egwuagu CE. Therapeutic Targeting of STAT3 (Signal Transducers and Activators of Transcription 3) Pathway Inhibits Experimental Autoimmune Uveitis. PloS One (2012) 7(1):e29742. 10.1371/journal.pone.0029742 22238646 PMC3252323

[78] Hilton DJ. Negative Regulators of Cytokine Signal Transduction. Cell Mol Life Sci (1999) 55(12):1568–77. 10.1007/s000180050396 PMC11146996 10526574

[79] Alexander WS. Suppressors of Cytokine Signalling (SOCS) in the Immune System. Nat Rev Immunol (2002) 2(6):410–6. 10.1038/nri818 12093007

[80] Alexander WS Hilton DJ. The Role of Suppressors of Cytokine Signaling (SOCS) Proteins in Regulation of the Immune Response. Annu Rev Immunol (2004) 22:503–29. 10.1146/annurev.immunol.22.091003.090312 15032587

[81] Yu CR Mahdi RR Oh HM Amadi-Obi A Levy-Clarke G Burton J. Suppressor of Cytokine Signaling-1 (SOCS1) Inhibits Lymphocyte Recruitment Into the Retina and Protects SOCS1 Transgenic Rats and Mice From Ocular Inflammation. Invest Ophthalmol Vis Sci (2011) 52(9):6978–86. 10.1167/iovs.11-7688 PMC3176005 21778271

[82] Starr R Willson TA Viney EM Murray LJ Rayner JR Jenkins BJ. A Family of Cytokine-Inducible Inhibitors of Signalling. Nature (1997) 387(6636):917–21. 10.1038/43206 9202125

[83] Flowers LO Johnson HM Mujtaba MG Ellis MR Haider SM Subramaniam PS. Characterization of a Peptide Inhibitor of Janus Kinase 2 That Mimics Suppressor of Cytokine Signaling 1 Function. J Immunol (2004) 172(12):7510–8. 10.4049/jimmunol.172.12.7510 15187130

[84] Waiboci LW Ahmed CM Mujtaba MG Flowers LO Martin JP Haider MI. Both the Suppressor of Cytokine Signaling 1 (SOCS-1) Kinase Inhibitory Region and SOCS-1 Mimetic Bind to JAK2 Autophosphorylation Site: Implications for the Development of a SOCS-1 Antagonist. J Immunol (2007) 178(8):5058–68. 10.4049/jimmunol.178.8.5058 17404288

[85] He C Yu CR Sun L Mahdi RM Larkin J,3 Egwuagu CE. Topical Administration of a Suppressor of Cytokine Signaling-1 (SOCS1) Mimetic Peptide Inhibits Ocular Inflammation and Mitigates Ocular Pathology During Mouse Uveitis. J Autoimmun (2015). 10.1016/j.jaut.2015.05.011 PMC4529792 26094775

[86] Luger D Silver PB Tang J Cua D Chen Z Iwakura Y. Either a Th17 or a Th1 Effector Response can Drive Autoimmunity: Conditions of Disease Induction Affect Dominant Effector Category. J Exp Med (2008) 205:799–810. 10.1084/jem.20071258 18391061 PMC2292220

[87] Amadi-Obi A Yu CR Dambuza I Kim SH Marrero B Egwuagu CE. Interleukin 27 Induces the Expression of Complement Factor H (CFH) in the Retina. PloS One (2012) 7(9):e45801. 10.1371/journal.pone.0045801 23029250 PMC3447806

[88] Lee YS Amadi-Obi A Yu CR Egwuagu CE. Retinal Cells Suppress Intraocular Inflammation (Uveitis) Through Production of interleukin-27 and Interleukin-10. Immunology (2011) 132(4):492–502. 10.1111/j.1365-2567.2010.03379.x 21294722 PMC3075503

[89] Collison LW Chaturvedi V Henderson AL Giacomin PR Guy C Bankoti J. Il-35-mediated Induction of a Potent Regulatory T Cell Population. Nat Immunol (2010) 11(12):1093–101. 10.1038/ni.1952 PMC3008395 20953201

[90] Wang RX Yu CR Dambuza IM Mahdi RM Dolinska MB Sergeev YV. Interleukin-35 Induces Regulatory B Cells That Suppress Autoimmune Disease. Nat Med (2014) 20(6):633–41. 10.1038/nm.3554 PMC4048323 24743305

[91] Choi JK Dambuza IM He C Yu CR Uche AN Mattapallil MJ. IL-12p35 Inhibits Neuroinflammation and Ameliorates Autoimmune Encephalomyelitis. Front Immunol (2017) 8:1258. 10.3389/fimmu.2017.01258 29051763 PMC5633738

[92] Dambuza IM He C Choi JK Yu CR Wang R Mattapallil MJ. Il-12p35 Induces Expansion of IL-10 and IL-35-expressing Regulatory B Cells and Ameliorates Autoimmune Disease. Nat Commun (2017) 8(1):719. 10.1038/s41467-017-00838-4 28959012 PMC5620058

[93] Kang M Choi JK Jittayasothorn Y Egwuagu CE. Interleukin 35-Producing Exosomes Suppress Neuroinflammation and Autoimmune Uveitis. Front Immunol (2020) 11:1051. 10.3389/fimmu.2020.01051 32547555 PMC7272665

